# Time-dependent degree-degree correlations in epileptic brain networks: from assortative to dissortative mixing

**DOI:** 10.3389/fnhum.2015.00462

**Published:** 2015-08-20

**Authors:** Christian Geier, Klaus Lehnertz, Stephan Bialonski

**Affiliations:** ^1^Department of Epileptology, University of BonnBonn, Germany; ^2^Helmholtz Institute for Radiation and Nuclear Physics, University of BonnBonn, Germany; ^3^Interdisciplinary Center for Complex Systems, University of BonnBonn, Germany; ^4^Max-Planck-Institute for the Physics of Complex SystemsDresden, Germany

**Keywords:** epileptic brain networks, EEG, assortativity, clustering coefficient, time-dependence, pre-seizure states, daily rhythms

## Abstract

We investigate the long-term evolution of degree-degree correlations (assortativity) in functional brain networks from epilepsy patients. Functional networks are derived from continuous multi-day, multi-channel electroencephalographic data, which capture a wide range of physiological and pathophysiological activities. In contrast to previous studies which all reported functional brain networks to be assortative on average, even in case of various neurological and neurodegenerative disorders, we observe large fluctuations in time-resolved degree-degree correlations ranging from assortative to dissortative mixing. Moreover, in some patients these fluctuations exhibit some periodic temporal structure which can be attributed, to a large extent, to daily rhythms. Relevant aspects of the epileptic process, particularly possible pre-seizure alterations, contribute marginally to the observed long-term fluctuations. Our findings suggest that physiological and pathophysiological activity may modify functional brain networks in a different and process-specific way. We evaluate factors that possibly influence the long-term evolution of degree-degree correlations.

## 1. Introduction

Over the past years, network theory has proven successful in characterizing interactions among the constituents of diverse complex systems, ranging from technological and biological to social systems (Albert and Barabási, 2002; Barabási and Oltvai, 2004; Boccaletti et al., 2006; Arenas et al., 2008; Bullmore and Sporns, 2009; Barabási et al., 2011; Barthélemy, 2011; Bashan et al., 2012; Holme and Saramäki, 2012; Newman, 2012; Stam and van Straaten, 2012; Borgatti et al., 2013; Csermely et al., 2013; Pessoa, 2014; Stam, 2014). In epileptology, the characterization of large-scale brain networks with concepts from network theory provides increasing evidence of seizure dynamics (generation, spread, and termination) within a network of brain regions (so called epileptic network), which generate and sustain normal, physiological brain dynamics during the seizure-free interval (Lehnertz et al., 2009; Richardson, 2010; Kramer and Cash, 2012; Terry et al., 2012; van Diessen et al., 2013; Lehnertz et al., 2014). Epilepsy–one of the most common neurological disorders with 50 million affected individuals worldwide (Duncan et al., 2006; Guerrini, 2006)—is now regarded as a network disease (Berg and Scheffer, 2011), i.e., a disease of functionally and/or structurally aberrant connections on virtually all spatial scales, from single neurons via groups of neurons to the systems level (Engel et al., 2013).

Improving our understanding of the emergence of epileptogenesis and ictogenesis from large-scale epileptic brain networks calls for approaches that take into account the interplay between the dynamic properties of network constituents (i.e., nodes and links) and the network topology. When investigating epileptic networks, nodes are usually assumed to represent distinct brain regions and links represent interactions between them, and these nodes and links constitute a functional brain network. Assessing the long-term dynamics of individual brain regions or nodes is mostly based on scalp or invasively recorded electroencephalograms, and links are derived from quantifying the statistical interdependence between signals (also referred to as functional connectivity) captured from electrodes overlying or within different brain regions (see Rubinov and Sporns, 2010; Lehnertz et al., 2014 for details). In the majority of network studies on epilepsy, global properties of epileptic networks during seizures have been characterized with measures such as clustering coefficient, average shortest path length, or synchronizability (see Lehnertz et al., 2014 for an overview). More recent studies explored the relevance of local network properties–such as the importance of individual nodes (Koschützki et al., 2005; Rubinov and Sporns, 2010)—for the dynamics of seizures (Kramer et al., 2008; Wilke et al., 2011; Varotto et al., 2012; Burns et al., 2014; Geier et al., 2015; Zubler et al., 2015). Findings achieved so far for these seizure networks are quite intriguing, given the similarity of their topological evolution across different types of epilepsies, seizures, medication, age, gender, and other clinical features, which might point to a common biophysical mechanism underlying ictogenesis. This similarity, however, is contrasted by strong intra- and interindividual fluctuations of local and global statistical network properties seen for the temporal evolution of epileptic brain networks over periods of days (Kuhnert et al., 2010; Kramer et al., 2011; Geier et al., 2013).

The structural and dynamic properties of networks can be deeply affected by the assortativity (also known as assortative mixing), the tendency of nodes with similar properties (e.g., number of links) to connect (Newman, 2002, 2003; Foster et al., 2010). Assortative mixing with respect to the number of links (i.e., node degrees) has been widely studied (Barrat et al., 2008). In this context, networks are called assortative if nodes connect preferentially with nodes of similar degree. If nodes connect preferentially with nodes of different degree, networks are called dissortative (or disassortative). Networks that are neither assortative nor dissortative are called degree-degree uncorrelated networks. Many technological, biological (such as structural brain networks, Bassett et al., 2008, 2011; Hagmann et al., 2008), and certain social networks (Holme et al., 2004; Fagiolo and Mastrorillo, 2013) are considered paradigmatic for a dissortative mixing (Newman, 2002, 2003). Various other social networks (Newman and Park, 2003; Croft et al., 2005; Bollen et al., 2011; Mac Carron and Kenna, 2013; Ke and Ahn, 2014), the cardiorespiratory interaction network (Long et al., 2014), protein contact networks (Bagler and Sinha, 2007), and particularly functional brain networks during normal physiological (Park et al., 2008; Jalili and Knyazeva, 2011; Schwarz and McGonigle, 2011; Braun et al., 2012) and during pathophysiological conditions (Bassett et al., 2008; de Haan et al., 2009; Wang et al., 2010; Kramer et al., 2011; Barzegaran et al., 2012; Agosta et al., 2013, 2014; Bialonski and Lehnertz, 2013) were reported to be assortative. Such networks are likely to have a comparatively resilient core of mutually interconnected high-degree nodes (Maslov et al., 2004) which makes them more robust against node removal (Newman, 2003; Vázquez and Moreno, 2003) and easier to synchronize (Motter et al., 2005; di Bernardo et al., 2007).

In Bialonski and Lehnertz (2013), functional brain networks before, during, and after one-hundred epileptic seizures with different anatomical onset locations were shown to exhibit assortative mixing patterns. Assortativity increased during seizures, reached a maximum prior to the end of seizures, and decreased already prior to seizure end. Interestingly, the observed concave-like temporal evolution of assortativity over the seizure period resembled closely the ones seen for the global correlation structure (Schindler et al., 2007), for the largest eigenvalue of adjacency matrices based on coherence and cross power (Müller et al., 2011), for clustering coefficient and average shortest path length (Schindler et al., 2008; Bialonski et al., 2011), and–if inverted–for synchronizability (Schindler et al., 2008). Taken together, these findings not only provide important clues on how the topology of functional brain networks changes during seizures but also on how seizures stop. In order to further improve our understanding of network mechanisms underlying seizure generation paralleling generation and maintenance of normal, physiological brain dynamics during the seizure-free interval, we here follow Kuhnert et al. (2010); Kramer et al. (2011); Geier et al. (2013) and investigate the long-term behavior of degree-degree correlations in evolving epileptic networks.

## 2. Materials and methods

### 2.1. Patient data

We analyzed long-term, multichannel invasive EEG (iEEG) recordings from seven patients who suffered from pharmacoresistant focal epilepsy with neocortical and/or hippocampal origin (cf. Table 1). After presurgical evaluation, all patients underwent resective surgery that led to complete seizure control. The patients had signed informed consent that their clinical data might be used and published for research purposes, and the study was approved by the ethics committee of the University of Bonn.

**Table 1:** **Clinical data of the patients**.

**ID**	**Age/Gender**	**dur**.	**foc. hem**.	**foc. reg**.	**szr. type**	**AED**	***N*_*rs*_**	***N*_*sz*_**	***d***
1	25/f	20	Right	Temporal-mesial	CPS	LEV, LTG	60	4	175
2	57/m	51	Right	Frontal	CPS	LEV, OXC, TPM	74	5	87
3	52/m	51	Left	Temporal-mesial	CPS	LEV, LTG	44	1	74
4	48/f	34	Right	Temporal-mesial	–	TPM	90	0	327
5	27/f	15	Left	Temporal-mesial	CPS, SG	LEV, LTG	50	2	144
6	13/m	1	Left	Parietal	–	LEV	64	0	100
7	37/m	4	Right	Temporal-mesial	CPS, SG	LTG	48	4	108

*ID, identification number; gender: f, female and m, male; age and duration (dur.) of epilepsy in years; foc. hem., focal hemisphere; foc. reg., focal region; seizure (szr.) type: CPS, complex partial seizures; SG, secondary generalized seizures; antiepileptic drugs (AED): LEV, levetiracetam; LTG; lamotrigine; OXC, oxcarbazepine; TPM, topiramate; N_rs_, number of recording sites; Nsz, number of seizures; d, duration of iEEG recording in hours*.

iEEG data were recorded continuously from chronically implanted intrahippocampal depth and/or subdural grid and strip electrodes (all manufactured by AD-TECH, WI, USA) using a Stellate Harmonie recording system (Stellate, Montreal, Canada; amplifiers constructed by Schwarzer GmbH, München, Germany). Decisions regarding electrode placement were purely clinically driven and were made independently of this study. The total number of electrode contacts ranged from *N*_rs_ = 44 to *N*_rs_ = 90. Data were band-pass filtered between 0.1 and 70 Hz, sampled at 200 Hz using a 16 bit analog-to-digital converter, and referenced against the average of two recording contacts outside the focal region. Reference contacts were chosen independently for each patient. The overall recording time amounted to 1015 h (range 74–327 h) during which 16 spontaneous seizures occurred (cf. Table 1). In each patient, antiepileptic medication was varied individually during the recording period.

### 2.2. Constructing functional networks and assessing their topological properties

#### 2.2.1. Defining nodes and links

Following Horstmann et al. (2010); Kuhnert et al. (2010, 2013), we constructed functional networks from iEEG data by associating network nodes with electrode contacts and inferred network links by estimating interdependencies between iEEG time series from pairs (*n, m*) (*n, m* ∈ {1, …, *N*_rs_}) of brain regions, regardless of their anatomical connectivity. For this purpose we used an established method for studying time-variant changes in phase synchronization, namely the mean phase coherence (Mormann et al., 2000):

(1) $$\begin{array}{l} R_{nm}=|\frac{1}{N}\overset{N-1}{\underset{j=0}{\sum }}\exp i\left(\Phi_{n}\left(j\right)-\Phi_{m}\left(j\right)\right)|, \end{array}$$

which is the temporal average of the differences of the instantaneous phases Φ of iEEG time series from nodes *n* and *m*, and *N* denotes the number of data points. By definition, *R*_*nm*_ is confined to the interval [0,1] where *R*_*nm*_ = 1 indicates fully phase-synchronized systems. We used the analytic signal approach (Gabor, 1946; Panter, 1965) and derived the instantaneous phases of an iEEG time series using the Hilbert transform. An important property of this approach is that the instantaneous frequency relates to the predominant frequency in the Fourier spectrum (Boashash, 1992; Frei et al., 2010), which may be subject to fluctuations in the iEEG time series. In such a case, the instantaneous frequency varies rhythmically around the predominant frequency resulting in spurious estimates of the instantaneous phase, which can, however, be reduced by taking the temporal average (cf. Equation R). From an electrophysiological point of view, we consider it more reasonable to look adaptively (via the Hilbert transform) at synchronization between predominant rhythms in the iEEG than to look at synchronization in some a priori fixed frequency bands for which there is no power in the time series (cf. Bruns, 2004; Osterhage et al., 2007; Frei et al., 2010).

#### 2.2.2. Deriving a temporal sequence of functional networks

For further analyses, we split the offline bandpass-filtered (1–45 Hz) iEEG time series into consecutive non-overlapping windows of 20.48 s duration each (corresponding to *N* = 4096 data points) and estimated, for each window, the elements *R*_*mn*_ of the phase synchronization matrix **R**. From this matrix, we constructed binary networks using a thresholding approach and set the non-diagonal elements of the adjacency matrix **A** to *A*_*mn*_ = 1 if the corresponding entry *R*_*mn*_ of **R** exceeded a threshold Θ, and to *A*_*mn*_ = 0 otherwise (*A*_*mm*_ = 0 ∀*m*). For each matrix **R**, we chose Θ such that the resulting network possessed a predefined link density (Anderson et al., 1999)

(2) $$\begin{array}{l} \rho =\frac{\bar{k}}{\left(N\text{rs}-1\right)}, \end{array}$$

with the number of nodes *N*_rs_ and the mean degree $\bar{k}=N_{{}_{\text{rs}}}^{-1}\sum_{n=1}^{N_{\text{rs}}}k_{n}(k_{n}:\text{​ }=\sum_{m=1}^{N_{\text{rs}}}A_{nm}$ denotes the degree of node *n*). To do so, we performed a rank ordering of the entries of the lower triangular part of *R* (excluding the main diagonal) and chose Θ as the (ρ*M*_*rs*_ − 1)-largest entry (with the number of all possible links *M_rs_* = (*N*^2^_rs_ − *N*_rs_)/2). We set ρ = 0.1 to define links. With the aforementioned steps of analysis, we derived a temporal sequence of functional brain networks during inter-ictal, peri-ictal, and ictal periods spanning several days for each patient.

#### 2.2.3. Assortativity

To assess degree-degree correlations in each functional network, we employed the assortativity coefficient *a* (Newman, 2002, 2003). It quantifies whether links of the network tend to connect nodes of similar degrees with each other (in which case the network is called assortative; *a* > 0) or whether links preferentially connect high-degree nodes with low-degree nodes (dissortative network; *a* < 0). This tendency can be quantified by assessing the correlation between the degrees of nodes at both ends of links. To simplify the implementation of the assortativity coefficient, we can reformulate it in terms of the degrees of nodes (see Appendix B in Bialonski and Lehnertz, 2013; see also Lehnertz et al., 2014) which then reads

(3) $$\begin{array}{l} a={\left(F_{1}F_{3}-F_{2}^{2}\right)}^{-1}\left(2F_{1}\overset{N\text{rs}}{\underset{n=1}{\sum }}\overset{n-1}{\underset{m=1}{\sum }}A_{nm}k_{n}k_{m}-F_{2}^{2}\right), \end{array}$$

where $F_{u}=\sum_{n=1}^{N_{\text{rs}}}k_{n}^{u}$. By definition, *a* is confined to the interval [−1, 1]. Positive (negative) values of *a* indicate an assortative (dissortative) network, while a value of zero is indicative of a degree-degree uncorrelated network.

The finite size (i.e., the finite number of nodes) of networks can induce degree-degree correlations even for network ensembles that are degree-degree uncorrelated by construction (Barrat et al., 2008). In order to account for such finite-size effects and to distinguish between these and genuine degree-degree correlations, we considered ensembles of Erdős-Rényi random networks. Erdős-Rényi network ensembles are degree-degree uncorrelated and well investigated in the literature on random graphs (Erdős and Rényi, 1959, 1960, 1961; Newman, 2002). For each patient, we created an ensemble of 20 Erdős-Rényi networks, having the same finite number of nodes *N*_rs_ and the same link density ρ as the functional networks. From these random network ensembles, we determined the mean value as well as the standard deviation of the assortativity coefficient. We consider values of the assortativity coefficient of functional networks that deviate more than one standard deviation from the mean value obtained for Erdős-Rényi networks to indicate genuine degree-degree correlations which cannot be explained by finite-size effects.

Results of previous studies pointed toward a coevolution of the assortativity coefficient (Bialonski and Lehnertz, 2013) and the clustering coefficient (Schindler et al., 2008) during seizures, which may indicate that assortative mixing comes along with or may be due to the transitivity of network links, a hypothesis which has been discussed for social networks (Newman and Park, 2003). In order to investigate whether such a coevolution can also be observed on longer time scales encompassing inter-ictal and peri-ictal periods, we determined—in addition to the assortativity coefficient—the clustering coefficient for each functional network.

#### 2.2.4. Clustering coefficient

The clustering coefficient *C* (Albert and Barabási, 2002; Boccaletti et al., 2006) characterizes the transitivity in networks. We here define the local clustering coefficient *C*_*n*_ of node *n* as

(4) $$C_{n}=\{\begin{array}{ll} \frac{1}{k_{n}(k_{n}-1)}\sum_{m,l}\left(A_{nl}A_{nm}A_{ml}\right) & \text{if }k_{n}>1\\ 0 & \text{if }k_{n}\in \{0,1\}, \end{array}$$

taking care of nodes with no or only one connection (i.e., *k*_*n*_ ∈ {0, 1}). By averaging *C*_*n*_ over all nodes, we obtained the clustering coefficient *C*. *C*_*n*_ and *C* are confined to the interval [0, 1] by definition.

## 3. Results

**Figure 1:**
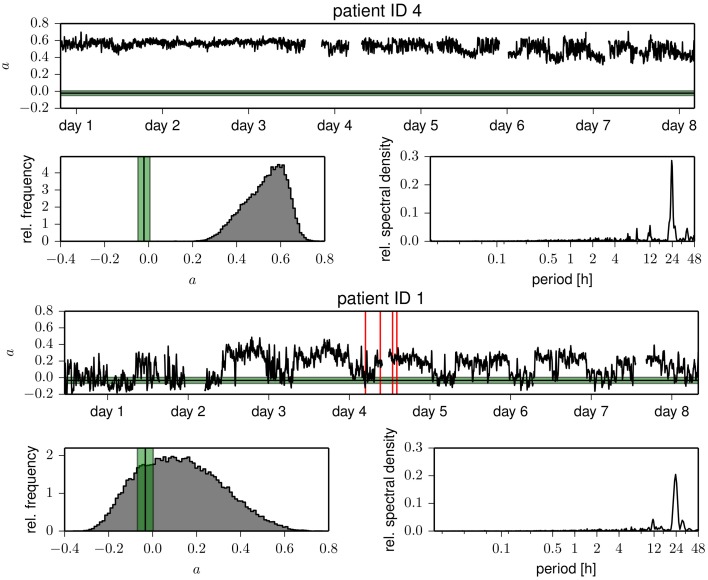
**Temporal evolutions of the assortativity coefficient of functional brain networks derived from patient 4 (upper part) and from patient 1 (lower part)**. Time profiles were smoothed using a moving average over 30 windows corresponding to 10.24 min for better legibility. Discontinuities are due to recording gaps. Tics on x-axes denote midnight. Vertical red lines mark the times of electrical onset of seizures, and the horizontal black lines (standard deviation is shown in green) denote the mean assortativity coefficient of Erdős-Rényi networks having the same number of nodes and the same link density as the functional networks. Below the time courses, we show the respective frequency distributions of the assortativity coefficient and power spectral density estimates of the temporal evolutions (Lomb-Scargle periodograms, computed by applying the algorithm proposed in Press and Rybicki, 1989 to the full, unfiltered, demeaned time profiles).

In Figure 1, we show time courses of the assortativity coefficient *a* derived from the temporal sequences of functional brain networks from two patients. We observe exclusively assortative mixing for patient 4, whereas for patient 1 repeated switches between phases of assortative and dissortative mixing can be observed (see Table 2 for the temporal means and their standard deviations of the assortativity coefficient for each patient). Of all patients, patient 1 was the only case for which we could observe long periods (up to several hours) of dissortative mixing. Shorter periods (up to several minutes) of dissortative mixing were observed in two other patients (patient 5 and 6). Since the number of recording sites *N*_*rs*_ (i.e., the size of a network) varied across patients, we checked whether this might have led to the large fluctuations seen in time-resolved degree-degree correlations but could not observe a clearcut relationship (the Pearson correlation coefficient between *N*_*rs*_ and mean assortativity coefficients amounted to 0.12).

**Table 2:** **Temporal means and standard deviations of assortativity coefficient ***a*** and clustering coefficient ***C*** for each patient**.

**ID**	***a***	***C***
1	0.13 ± 0.19	0.55 ± 0.06
2	0.39 ± 0.10	0.55 ± 0.04
3	0.47 ± 0.14	0.45 ± 0.06
4	0.53 ± 0.09	0.53 ± 0.03
5	0.16 ± 0.16	0.55 ± 0.04
6	0.20 ± 0.10	0.46 ± 0.04
7	0.49 ± 0.11	0.42 ± 0.05

The evolutions of the assortativity coefficients shown in Figure 1 exhibit large fluctuations over time and appear to be partly periodic. The power spectral density estimates point to strong contributions from processes acting on timescales of some tens of hours but only small contributions from processes acting on timescales less than 30 min. For four patients, we observe a strong component at about 24 h with less pronounced (about a factor of 8 and more) contributions at the subharmonics at about 12 and 8 h. This may point toward an influence of daily rhythms, while components of even longer time scales might be related to alterations of antiepileptic medication during the presurgical evaluation. Not only do we observe large intra- and interindividual fluctuations for the assortativity coefficient *a*, but even the extent of the intraindividual fluctuations is very different (see the standard deviations of *a* in Table 2).

**Figure 2:**
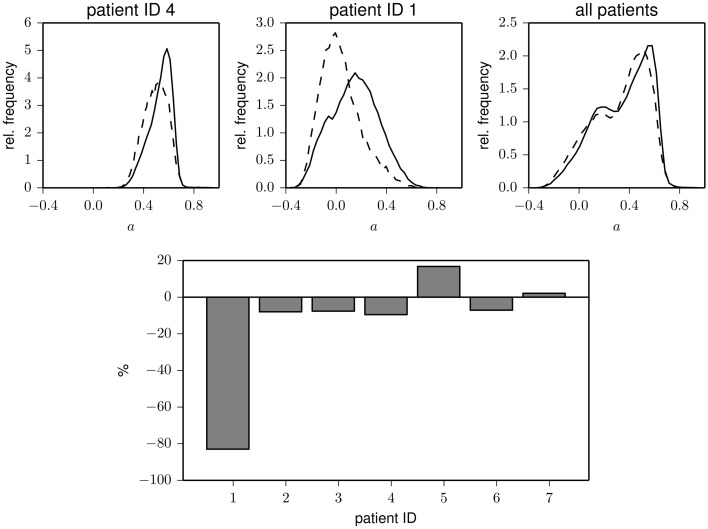
**Top:** Exemplary frequency distributions of the assortativity coefficient derived from data recorded during day (solid) and night times (dashed) for two patients (left and middle) and for the pooled data from all patients (right). **Bottom:** Relative changes of assortativity (*ā*_night_ − *ā*_day_)/*ā*_day_ during day and night times for each patient. *ā*_day_ and *ā*_night_ denote median values of the respective distributions.

Interestingly, the time courses shown in Figure 1 indicate that less assortative (or even dissortative) mixing can be observed preferentially during night times. In order to investigate whether this observation extends beyond exemplary data, we split the data recorded during night times (ranging from 22 p.m. to 6 a.m.) and during day times (ranging from 6 a.m. to 22 p.m.). In the upper part of Figure 2, we show distributions of the assortativity coefficient for patients 1 and 4 as well as for the pooled data from all patients. Given the large interindividual variability, differences between distributions are diverse (see lower part of Figure 2), but a preferentially less assortative mixing during night times can be observed for the group of patients investigated here.

Having identified daily rhythms as a potential factor that may strongly influence the long-term evolution of degree-degree correlations in functional epileptic networks, we next evaluated whether relevant aspects of the epileptic process, particularly possible pre-seizure alterations, contribute to the observed long-term fluctuations. Following Mormann et al. (2003); Le Van Quyen et al. (2005); Mormann et al. (2005); Schulze-Bonhage et al. (2006); Feldwisch-Drentrup et al. (2011); Lehnertz and Dickten (2015), we assumed that a pre-ictal phase of 4 h duration exists (Mormann et al., 2007) and compared the distributions of values of the assortativity coefficient from the pre-ictal periods with those from inter-ictal periods. The latter distribution included all data that were recorded at least 4 h prior to and 30 min after a seizure.

**Figure 3:**
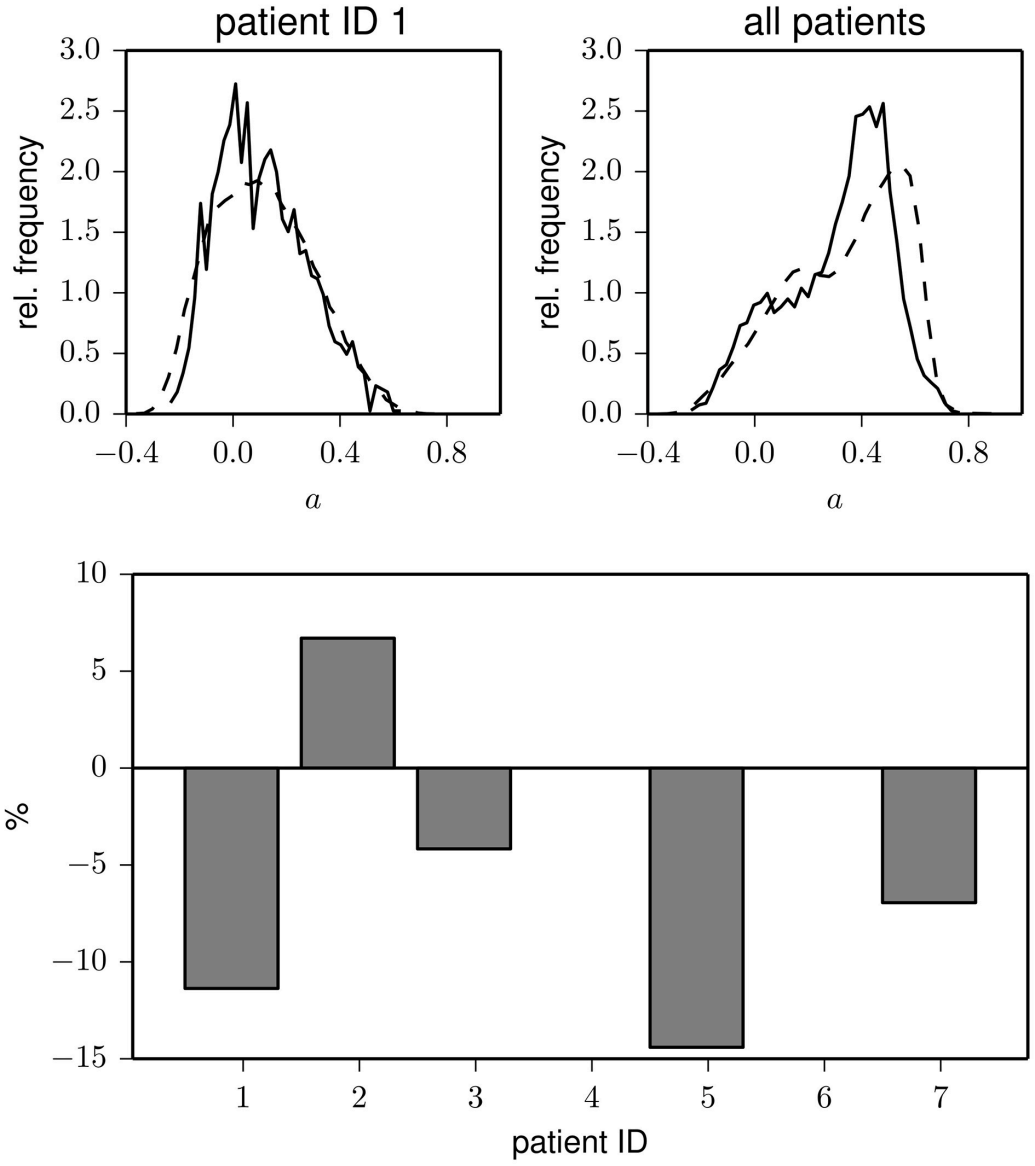
**Top:** Frequency distributions of the assortativity coefficient derived from data recorded during pre-ictal (solid) and inter-ictal periods (dashed) for one patients (left) and for the pooled data from all patients (right). **Bottom:** Relative changes of assortativity (*ā*_pre_ − *ā*_inter_)/*ā*_inter_ during pre-ictal and inter-ictal periods for each patient (patients 4 and 6 had no seizures during the recording period). *ā*_inter_ and *ā*_pre_ denote median values of the respective distributions.

In the upper part of Figure 3, we show the distribution of the assortativity coefficient for data from the pre-ictal and inter-ictal period from patient 1 as well as for the pooled data from all patients. Interestingly, the pre-ictal phase appears to be characterized by a slightly (about 10%) decreased assortative mixing, and in only one patient, we could observe a pre-ictal increase of degree-degree correlations (cf. lower part of Figure 3). Although these findings may help to further improve the understanding on how and which network reconfigurations promote seizure generation, more sophisticated analysis techniques (Andrzejak et al., 2009) applied to a larger dataset would be needed in order to statistically judge the observed pre-ictal changes.

**Figure 4:**
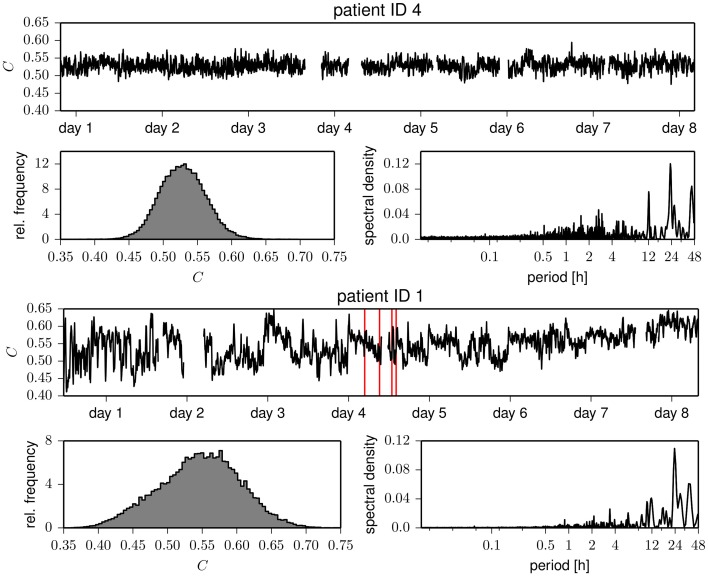
**Same as Figure 1 but for the clustering coefficient ***C*****.

**Figure 5:**
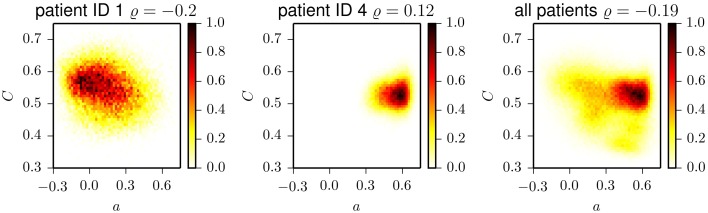
**Two-dimensional histograms of the frequencies of occurrence of pairs (***a, C***) for patient 1 (left), patient 4 (middle), and aggregated for all patients (right)**. Histograms are normalized to the maximum bin count. ϱ denotes the Pearson correlation coefficient which we determined for the respective datasets.

Eventually, we studied whether temporal changes in assortative mixing are correlated with temporal changes in network transitivity (as quantified by the clustering coefficient). The temporal evolutions of the clustering coefficient for exemplary patients are shown in Figure 4. Similar to the case of the assortativity coefficient, the time courses of the clustering coefficient show large fluctuations and periodic structures. These periodicities act on timescales of about 24 h, with less pronounced contributions from subharmonics at about 12 and 8 h, which confirms previous results (Kuhnert et al., 2010). The intra- and interindividual fluctuations of the clustering coefficient (cf. Table 2) are much less pronounced than the fluctuation of the assortativity coefficient. We only observed weak correlations between assortativity and clustering coefficient (as quantified by the Pearson correlation coefficient ϱ; cf. Figure 5) in five patients (Pearson correlation coefficient ranged from −0.20 to 0.28), while in two patients, the correlation vanished (|ϱ| ≪ 0.1). These results indicate that for the functional brain networks investigated here, the assortativity coefficient provides information about the long-term evolution of the functional brain networks, which is complementary to the information provided by the clustering coefficient.

## 4. Discussion

We investigated the long-term variability of degree-degree correlations (assortativity) of functional brain networks constructed from invasive EEG recordings from seven patients suffering from focal epilepsies with neocortical and/or hippocampal origin. We observed large fluctuations in time-resolved degree-degree correlations, ranging from dissortative to assortative functional brain networks. For all patients, we observed the temporal evolution of the assortativity coefficient to possess periodic structures. Power spectral densities of the temporal evolutions were dominated by contributions at a time scale of some tens of hours, which may point toward a potential influence of daily rhythms, and/or to changes of the antiepileptic medication. Potential processes related to much smaller time scales (< 30 min) contributed much less to the overall power, a finding also observed in a previous study (Kuhnert et al., 2010) for the clustering coefficient and the average shortest path length. In the majority of patients (five of seven patients), functional brain networks tended to show higher degree-degree correlations during day times than during night times. However, we also observed the opposite behavior for two patients. As there was no sleep scoring available, we cannot relate the data to specific sleep stages. Still, the overall trend indicates that assortativity seems to be reduced during night times.

Only small differences in degree-degree correlations could be found between functional brain networks during inter-ictal periods and those during periods of an assumed pre-ictal state. While we observed a pre-ictal decrease of the assortativity in four patients, we also observed a pre-ictal increase in assortativity for one patient (no seizures were recorded for two patients). It remains to be shown whether the observed tendency of functional brain networks to show slightly less assortative mixing pre-ictally can be regarded as predictive of the extreme event epileptic seizure.

**Figure 6:**
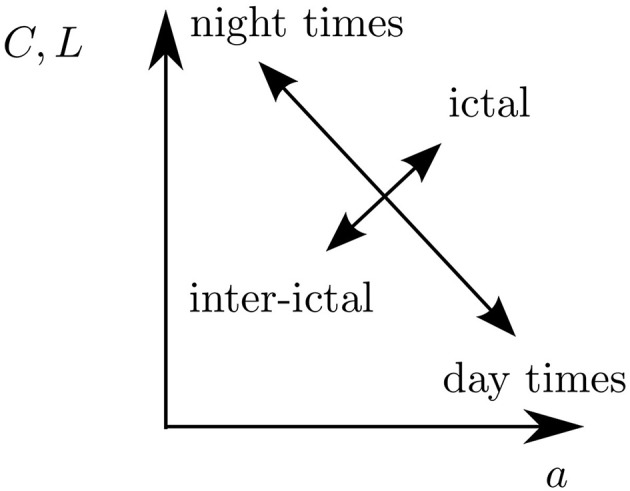
**Sketch of how functional brain networks may explore the space of accessible network topologies (here parametrized by the clustering coefficient ***C***, average shortest path length ***L***, and assortativity coefficient ***a***) in a process-dependent way:** Daily rhythms may be reflected in periodic reorganizations of functional brain networks. Superimposed on that may be a reorganization in functional network connectivity reflecting pathophysiological activity.

In the light of results reported in previous studies (Ponten et al., 2007; Schindler et al., 2008; Kramer et al., 2010, 2011; Kuhnert et al., 2010; Bialonski et al., 2011; Bialonski and Lehnertz, 2013), our findings might point toward the following: We speculate that daily rhythms may be reflected in periodic reorganizations of functional brain networks. Superimposed on that may be a reorganization in functional network connectivity reflecting pathophysiological activity. The space of accessible network topologies, however, may be explored in a different and process-dependent way (cf. Figure 6).

Changes due to daily rhythms seem to be reflected in functional brain networks, which have a larger transitivity and a larger average shortest path length (Kuhnert et al., 2010) but show less assortative mixing (as observed here) during night times than during day times. In contrast, as reported in previous studies, changes related to ictal activity seem to be associated with functional brain networks which are characterized by a larger transitivity, larger average shortest path length (Ponten et al., 2007; Schindler et al., 2008; Kramer et al., 2010; Bialonski et al., 2011), and more assortative mixing (Bialonski and Lehnertz, 2013) during seizures than before or after seizures. These latter changes suggest that functional brain networks might be more segregated during than before or after seizures (Kramer et al., 2010; Bialonski and Lehnertz, 2013): Nodes form different groups (Kramer et al., 2010) in which they share a similar node degree (leading to an increased assortativity, Bialonski and Lehnertz, 2013) and are tightly connected to each other (leading to an increased transitivity, Ponten et al., 2007; Schindler et al., 2008; Kramer et al., 2010; Bialonski et al., 2011). Between these groups, only sparse connections exist (leading to an increased average shortest path length, Ponten et al., 2007; Schindler et al., 2008; Kramer et al., 2010; Bialonski et al., 2011) which, in turn, may weaken the synchronizability of a network as a whole as suggested in previous studies (Schindler et al., 2008; Bialonski and Lehnertz, 2013). Changes in functional brain networks related to daily rhythms, however, appear to be expressed in a different way: During day times, nodes of the networks may be well connected (leading to a decreased average shortest path lengths, Kuhnert et al., 2010) via a few hub-like structures (i.e., nodes with high degrees; leading to an increased assortativity as observed here) which integrate information from all over the network. Individual nodes of lower degrees tend to be interconnected only sparsely (leading to a decreased clustering coefficient, Kuhnert et al., 2010). During night times, the hub-like structures disappear or are less pronounced (leading to a decreased assortativity as observed here), giving way to a segregation of groups (associated with an increased average shortest path length, Kuhnert et al., 2010) in which nodes are mutually well interconnected (leading to an increased clustering coefficient, Kuhnert et al., 2010).

We note that these differences between changes of network topologies related to physiological and pathophysiological activities only become apparent when considering degree-degree correlations, in addition to the previously investigated network measures (cf. Figure 6). This underlines the usefulness of characterizing functional brain networks using different network measures, including assortativity. It remains to be shown whether the differences in network changes according to physiological and pathophysiological activity can also be observed in a larger sample of patients (which will allow for a better statistics).

We consider future research as promising which aims at a more detailed characterization of time evolving functional brain networks. For instance, if these networks indeed possess a more pronounced hub-like structure during day than during night times, we speculate that this will likely be reflected in respective changes in the distributions of betweenness centralities. Hubs that appear during day times and which integrate information from most of the other nodes may be identifiable by the largest betweenness centralities that occur in a network.

Another important aspect that is not yet fully explored is the influence of changes of the antiepileptic medication. Altered drug levels appear to affect functional brain networks only locally (Lehnertz and Elger, 1997; Haneef et al., 2015), but it is not clear if and how they might lead to local and/or global network reorganizations. Eventually, it is in general a challenging and non-trivial problem how to relate local to global network properties. Thus, knowing whether and how exactly the reported periodic changes and reorganizations of functional brain networks can be related to features of the node and/or link dynamics may help to gain deeper insights into the complicated dynamics underlying epileptic networks.

## Funding

SB and KL acknowledge support by the Volkswagen Foundation (Grants No. 85390 and No. 85392).

### Conflict of interest statement

The authors declare that the research was conducted in the absence of any commercial or financial relationships that could be construed as a potential conflict of interest.
